# Exploratory development of biomarkers for neurobehavioral performance impairment during sleep loss: comparison across multiple types of sleep deprivation

**DOI:** 10.1186/s12864-025-12193-6

**Published:** 2025-11-14

**Authors:** Hilary A. Uyhelji, Scott J. Nicholson, Thomas E. Nesthus, Julia L. Beckel, Elizabeth B. Klerman, Charles A. Czeisler, Robin K. Yuan, Arturo Arrona-Palacios, Pamela Song, Joseph M. Ronda, Michael S. Goodson

**Affiliations:** 1 Federal Aviation Administration, Civil Aerospace Medical Institute, Oklahoma City, OK USA; 2 Division of Sleep and Circadian Disorders, Department of Medicine, Brigham and Women’s Hospital, Mass General Brigham, Boston, MA USA; 3 Division of Sleep Medicine, Harvard Medical School, Boston, MA USA; 4 Department of Neurology, Inje University Ilsan Paik Hospital, Inje University College of Medicine, Goyang, South Korea; 5 United States Air Force Research Laboratory, Wright Patterson Air Force Base, Dayton, OH USA

**Keywords:** Total sleep deprivation, Sleep restriction, Shiftwork, Neurobehavioral performance impairment, Fatigue, Psychomotor vigilance test, Gene expression biomarkers

## Abstract

**Background:**

Inter-individual variation in response to insufficient or altered timing of sleep presents a challenge for the development of personalized approaches to fatigue monitoring and mitigation. Insufficient sleep has adverse health impacts, can result in impaired neurobehavioral performance, and can substantially increase the risk of injury and even mortality in safety–critical operations such as transportation. The present study provides a detailed exploration of physiological, neurobehavioral, and gene expression changes during sleep restriction, acute total sleep deprivation, and altered timing of sleep among 59 healthy volunteer participants who completed a 10-day inpatient study.

**Results:**

Reducing the quantity or altering the timing of sleep significantly impacts self-reported estimates of sleep duration, polysomnography-recorded sleep stages, and neurobehavioral performance test results. Impaired neurobehavioral performance was associated with transcriptomic changes in gene expression. A comparison of current and prior research on total sleep deprivation indicated that reproducible candidate gene expression biomarkers exist for at least one metric of attention, specifically, Psychomotor Vigilance Test (PVT) lapses.

**Conclusions:**

Candidate biomarkers of fatigue-related impairment were identified that responded to single neurobehavioral performance endpoints, as well as those that responded to multiple types or metrics of performance. Reproducible identification of biomarker candidates for PVT lapses during total sleep deprivation increases confidence in the ability to develop a molecular approach to fatigue-related impairment detection, while novel discoveries expanded the list of candidate genes to other impairment metrics. Molecular biomarkers for neurobehavioral performance impairment represent a potentially valuable tool to more precisely monitor the neurobehavioral performance deficits resulting from sleep loss, and further research and validation could eventually augment fatigue risk management practices that historically emphasize scheduling and rest opportunities. The data generated from self-assessment, polysomnography, neurobehavioral performance, and molecular investigations provide a wealth of information made publicly available for further data mining and scientific advancements.

**Trial registration:**

ClinicalTrials.gov, TRN: NCT04211506, Registered 23 December 2019.

**Supplementary Information:**

The online version contains supplementary material available at 10.1186/s12864-025-12193-6.

## Introduction

Sleep quality and quantity are integral components of health, well-being, and performance. Consensus recommendations from the Sleep Research Society and American Academy of Sleep Medicine suggest a minimum of 7 h of nightly sleep for adults to optimize health [1]. Inadequate sleep is associated with multiple disease and cardiovascular risks, such as hypertension, endothelial dysfunction, cardiac arrythmias, coronary artery disease, myocardial infarction, and stroke [2–5]. Adequate sleep is important for improving individuals’ subjective sense of well-being and mental health [2, 6–8]. Numerous studies have documented associations of sleep with athletic, motor, and neurobehavioral performance [9–13].

Performance impairments associated with inadequate sleep pose substantial costs to the global economy and a serious risk of injury and mortality in safety–critical operations such as transportation. Annual workplace costs from sleep deficiencies have been estimated at $322–$1967 per employee [14], and a loss of $280–$411 billion to the United States economy [15]. In Canada, evidence suggests that a 5% improvement in sleep quantity among adults could result in cost-savings of $148 million annually [16]. Beyond financial costs, impaired performance from insufficient sleep in the workplace poses safety risks. Over a 12-year period, approximately 20% of major United States National Transportation Safety Board investigations identified fatigue as a significant contributing factor, a probable causal contributing factor, or a finding across transportation modes (23% in aviation) [17]. Insufficient sleep quality or quantity can result from a variety of causes, such as lifestyle choices and work requirements or schedules. Recognition by the Federal Aviation Administration of the risks to the national aerospace from reduced sleep quantity or circadian disruption due to shiftwork resulted in a recent external review and recommended changes to scheduling practices for the agency’s air traffic controllers [18].

One of the greatest challenges to managing the risks of inadequate sleep is the varied response of individuals to sleep loss or altered sleep timing. Two persons with identical sleep schedules may have different levels of performance impairment. Over the past decades, studies have demonstrated the potential for heritable, trait-like inter-individual variation in the response to sleep loss [19–23]. Research has begun to identify molecular variants potentially associated with inherited susceptibility or resistance to sleep disruption or loss [24, 25]. In addition, new lines of research such as the present study are exploring different types of molecular biological indicators (i.e., molecular biomarkers) to track current, transient fatigue-related impairment status based on activity levels of genes. By way of analogy, gene expression levels may change in association with physiological experiences, much as blood sugar levels may change following meal consumption. Studies of total sleep deprivation identified candidate ribonucleic acid (RNA) biomarkers consisting of genes differentially expressed in association with Psychomotor Vigilance Test (PVT) attention lapses during sleep loss [26, 27]. A panel-based test of such RNA biomarkers could serve as a metric to guide the monitoring of fatigue risks in safety–critical operations and help identify potential fatigue impairment during accident or incident investigations. In addition to gold-standard polysomnographic evaluations of sleep, the present study explored RNA gene expression changes associated with a wide range of objective neurobehavioral performance changes during acute total sleep deprivation, multiple nights of sleep restriction, and sleep restriction with daytime sleep among healthy adults.

## Methods

### Study design

All participants provided written informed consent with Institutional Review Board approval by Mass General Brigham/Brigham and Women’s Hospital (BWH) and the FAA Civil Aerospace Medical Institute (CAMI), with protection under an NIH Certificate of Confidentiality (CC-OD-21–2237) with ClinicalTrials.gov ID NCT04211506.

Intensive outpatient screening of participants was conducted to verify they were healthy, free of medication and substance use, and abstaining from caffeine and other stimulants. Recruitment targeted adults 20–45 years old with a body mass index of 18.5–29.9 kg/m^2^. Potential participants had a physical exam, review of self-reported medical history, and psychological screening [28]; those with reported personal or immediate family history of psychiatric disorders were excluded. Self-reported habitual sleep durations of 7–9 h nightly were required, and potential participants were excluded if they reported recent frequent night shift work (e.g., working > 3 nights per week between 01:00–06:00 h within the past 3 months). Potential participants at higher risk of sleep disorders were not enrolled, based on scores on the Athens Insomnia Scale [29, 30], Berlin Questionnaire for Obstructive Sleep Apnea [31], and a 5-question assessment for Restless Leg Syndrome. A home sleep test was conducted with a Nox-T3 portable monitor (Nox-T3, Nox Medical, Reykjavik, Iceland), and potential participants were excluded if results indicated an apnea–hypopnea index of ≥ 15 or a periodic limb movement index ≥ 20. Finally, potential participants representing extreme morning or evening chronotypes were excluded based on scores < 31 (evening type) or ≥ 69 (morning type) on the Horne-Östberg Morningness-Eveningness Questionnaire [32].

To reduce the potential for recent outpatient sleep loss to influence study results, immediately prior to beginning the 10-day inpatient study all participants were asked to complete at least 1 week with 8 h of Time In Bed (TIB) each night followed by at least 1 week of 10 h TIB each night. Adherence was monitored via actigraphy (CamNTech Motion Watch 8), time-stamped call-ins at bedtime and wake time, and a daily sleep diary.

Participants passing outpatient screening procedures were admitted to the Center for Clinical Investigation at the Brigham and Women’s Hospital for the inpatient 10-day study. Throughout the study, the participant lived in a private study room complete with bed, bathroom, and computers for testing. The study rooms were designed to minimize noise and light from adjacent areas of the facility and were maintained at temperatures approximately 71–77 degrees Fahrenheit. Fluorescent room lighting targeting intensities < 100 lx was controlled by the investigators with overhead fixtures during scheduled time awake, and all room lights were turned out during TIB. There were no windows in the study rooms. To minimize external influences during the inpatient study, participants were blinded to their group assignment and knowledge of time of day was restricted, as was access to clocks, the internet, live television or radio, and contact with non-hospital personnel. After the first two acclimation days with ad lib meals, meals typically were provided every 4 h during time awake in accordance with a weight-maintenance diet based on anticipated caloric need as calculated using the Mifflin St. Jeor equation (activity factor 1.6) [33].

**Fig. 1:**
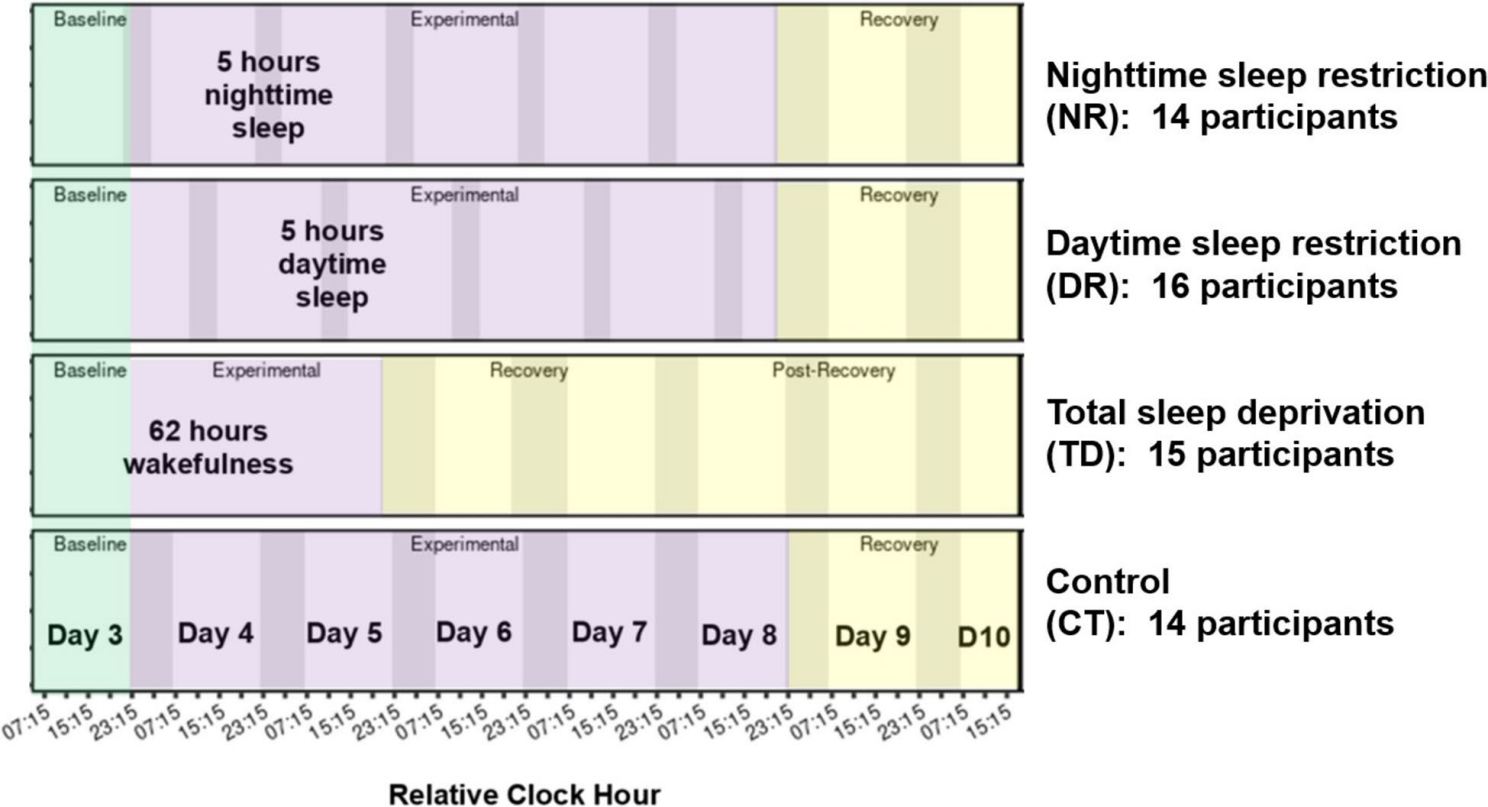
Schematic of study design. Overview of schedule for inpatient study day 3–10, with darker shading indicating scheduled sleep Time In Bed (TIB) and colors reflecting baseline (green), experimental (purple), and recovery (yellow) study segments. Neurobehavioral performance test batteries were conducted every 4 h except during scheduled TIB, with the approximately 45-min test battery beginning at the Relative Clock Hours indicated with tick marks on the x-axis (i.e., 03:15, 07:15, 11:15, 15:15, 19:15, and 23:15 h)

A total of 73 participants began the inpatient study, and 59 completed all 10 days. Participants were randomized without replacement to one of four study conditions that differed in the experimental segment treatment: a control with 8 h TIB nightly (CT, 14 participants), sleep restriction with 5 h TIB during daytime across 5 days (DR, 16 participants), sleep restriction with 5 h TIB during nighttime across 5 nights (NR, 14 participants), and acute total sleep deprivation with no sleep across two nights (TD, 15 participants) (Fig. 1). In all four study conditions, participants began with an acclimation segment on the day of admission (day 1), a nighttime sleep opportunity with 12 h TIB, a second acclimation day, and then 8 h TIB. This was followed by a baseline day (day 3), the experimental segment (CT, DR, NR, or TD treatment), and finally ended with a recovery segment incorporating 8–10 h TIB prior to discharge. Participants were continuously monitored during the inpatient phase to improve compliance, including onsite technicians who would enter the room if a patient failed to respond to tasks or as needed. Technicians also frequently entered the room for reasons such as sample collection and meal delivery.

All times reported are based on Relative Clock Hour (RCH), with the actual time of scheduled inpatient study events adjusted for each participant based on the average midpoint of their outpatient self-reported sleep–wake schedule. Time intervals between scheduled events were the same for all participants in each of the four condition groups (Additional file 1: Table S1); for example, neurobehavioral test batteries were scheduled 4 h apart during time awake. However, events such as the 08:00 (RCH) blood draw on baseline day 3 might occur at slightly different real-world actual clock times across participants.

### Sleep and neurobehavioral performance monitoring

During the night before baseline day 3 and during the two recovery nights after the experimental segment of DR, NR, and TD conditions (for CT, the final two study nights), polysomnography (PSG) monitoring was conducted using the Vitaport digital ambulatory EEG recording system (Vitaport-3, TEMEC Technologies B.V., Heerlen, The Netherlands). The PSG recording involved electroencephalogram (EEG), bilateral electrooculogram (EOG), chin electromyogram (EMG; mentalis, submentalis), and two-lead electrocardiogram (ECG). Visual scoring was conducted by a registered PSG technician following the American Academy of Sleep Medicine criteria version 2.6 [34]. In addition, within approximately 5–10 min of the conclusion of each TIB sleep opportunity, participants provided self-assessments of their prior night’s sleep and estimated total sleep duration.

At 4-h intervals during time awake from baseline day 3 until discharge from the inpatient study, participants completed an approximately 45-min computer-based neurobehavioral performance test battery of objective and subjective tests. Participants performed approximately 3–5 practice tests during the first two days of acclimation to the inpatient study (not analyzed), which were intended to reduce learning effects during data collection days 3–10. Subjective assessments were conducted using scales such as the Karolinska Sleepiness Scale (KSS) [35], a 9-point Likert scale of sleepiness. Another subjective element was the Performance Effort and Evaluation Rating Scale (PEERS) [36], consisting of responses on a Visual Analog Scale (VAS) rating their estimate of how well they performed on the neurobehavioral test battery (from extremely good to poor); how much effort they had to expend; and whether they could have performed better if they had tried harder. Participants also provided VAS responses on bipolar visual analog scales rating their feelings of being alert-sleepy, calm-excited, happy-sad, groggy-clearheaded, and unmotivated-motivated [37].

The neurobehavioral performance test battery also encompassed objective evaluations, such as the 90-s version of the Digit Symbol Substitution Test (DSST). The DSST gauges cognitive function by requiring participants to match symbols with corresponding digits [38]. Another assay was the Stroop color-word test (STROOP) [39] of executive functioning or reasoning, during which participants were asked to respond to font color while ignoring a written word (which spelled one of four colors). Due to the unintentional study enrollment of one red-green color-blind participant, STROOP data were analyzed in two ways: once with all four colors omitting this participant, and again for all participants based on only portions of the test with blue-yellow colors. In another test called the Matrix Reasoning Test (MRT), problem-solving skills were evaluated on trials of varying difficulty. For this, participants were required to determine which matrix completes a set of eight other matrices based on 1–3 relational pattern changes or transformations [40]. Memory and recall were tested via the Face-name task (FACE), in which participants were asked to recall face-name pairs presented approximately 33 min previously [41]. Attention was assessed with the 10-min Psychomotor Vigilance Test (PVT) of reaction time, encompassing measures of the speed of response and lapses (i.e., failure to respond to a stimulus within 0.5 s) [42]. Spatial coordination was assayed with the Unstable Track assay (TRACK), in which participants attempted to keep a moving cursor in the middle of the screen between two vertical lines [43]. Spatial orientation and response to feedback stimuli were probed with the Comparative Visual Search (CVS) [44], in which participants scanned the computer display for a mismatch between copy or mirror images consisting of distributions of right and left-oriented triangles. Finally, a risk-taking assessment was performed with the Balloon Analog Risk Task (BART), during which participants were asked to maximize their reward by choosing the number of times to inflate an animated balloon, potentially increasing their artificial reward with each inflation at the risk of loss if the balloon popped [45].

From the 59 participants who completed the study (see Results), there were a total of 2039 neurobehavioral performance test battery data collections attempted every 4 h during time awake on study days 3–10. Subdivided among the 4 study condition groups, this consisted of 434 data collections attempted for CT (14 participants, 31 timepoints each), 576 for DR (16 participants; 36 timepoints each), 504 for NR (14 participants, 36 timepoints each), and 525 for TD (15 participants, 35 timepoints). A small number of data collections were omitted from final analysis due to technical issues, responses omitted by the subject, or otherwise deemed unreliable. The most omissions were for the STROOP due to one subject seeming to have misunderstood the instructions and a second who was colorblind, resulting in 39 (results on blue-yellow tests only) to 75 (all colors) timepoints being omitted. For other neurobehavioral performance test battery endpoints, fewer than 10 of the 2039 possible timepoints were omitted. Neurobehavioral performance test battery results and data dictionary descriptions (including specification of omitted timepoints) have been posted for public access at the National Center for Biotechnology Information database of genotypes and phenotypes (see Availability of Data and Materials).

### Sample collection and sequencing

Whole blood consisting of a 2.5 mL draw was collected into PAXgene® Blood RNA tubes (BD Biosciences Catalog No. 762165) during inpatient study days 3–10, with approximately 26–31 timepoints per participant. For all participants blood draws began in the morning of inpatient study day 3 (baseline segment) at 08:00 (RCH) (Additional file 1: Table S1). For CT participants, draws continued every 4 h during time awake through 16:00 (RCH) prior to departure on day 10, for a total of 31 draws. For TD participants, blood draws continued every 4 h during time awake through 20:00 (RCH) on study day 7 (recovery segment), followed by single timepoint morning blood draws at 08:00 (RCH) on study days 8–10, for a total of 27 draws. For NR and DR participants, blood draws continued every 4 h during time awake for study days 3–4, 6, and 8–10, for a total of 26 draws. Blood was not drawn during study days 5 and 7 for NR and DR participants. For all participants, after each draw the PAXgene® Blood RNA tube was gently inverted by hand 10 times, allowed to sit at room temperature for approximately 6 h, frozen at −20 degrees Celsius for approximately 25 h, and then transferred to −80 degrees Celsius until extraction. Blood draws were taken via intravenous lines immediately following the neurobehavioral performance test battery and (typically) preceding a meal. Study staff were trained such that in the rare event of a failed collection with the intravenous line draw, the blood draw could be attempted again with a butterfly needle (not analyzed).

Total ribonucleic acid (RNA) was extracted from PAXgene® Blood RNA tubes by either the FAA Civil Aerospace Medical Institute (CAMI) or the Baylor College of Medicine Human Genome Sequencing Center (Baylor). Extractions by CAMI were performed on approximately three samples per participant (roughly ten percent) for periodic quality checks of sample material prior to submission of the remaining sample set to Baylor. Timepoints for CAMI extractions were selected in a stratified pseudo-random fashion. The CAMI extractions were conducted on the QIAcube Connect with an automated spin-column approach using the PAXGene Blood miRNA kit (Qiagen 763134), with final RNA elution into nuclease-free water. The remaining samples were extracted by Baylor using the Chemagic Prime Total RNA Blood 4 k kit (PerkinElmer, catalog #CMG-1484) and the Magnetic Bead technology Chemagic Prime 8 platform. Baylor prepared libraries from all RNA (i.e., both samples extracted by Baylor and CAMI) using the Illumina TruSeq Stranded Total RNA with Ribo-Zero Globin kit, followed by sequencing targeting 100 million forward plus reverse 150 base pair paired-end reads, as previously described [26]. Sequences were analyzed by CAMI, as described below.

### Analyses

Phenotypic data exploration involved the generation of plots in CRAN R versions 4.3.3 and R version 4.4.1 [46], with ggplot2 version 3.5.1 [47], and model runs to test for associations with the study condition groups. For metrics in the 45-min neurobehavioral performance test battery, linear models were run with the lmerTest package [48] version 3.1–3 command ‘lmer’ if qq-plots showed an approximately normal distribution (default settings, with fit by REML and t-tests using Satterthwaite’s method). Otherwise generalized linear models in the lme4 package version 1.1–35.5 [49] were run with the glmer command using Gamma (continuous) or Poisson (count) distributions, log link, default settings, and fit by maximum likelihood (Laplace Approximation). Other packages used included psych package version 2.4.6.26 [50], tidyverse version 2.0.0 [51], gtools version 3.9.5 [52], dplyr [53], and optimx 2023–10.21 [54]. Models contained a random term for the participant and fixed terms for the study condition group as well as for the cumulative number of hours since midnight (RCH) at the outset of baseline study day 3. This cumulative hours covariate was centered and scaled with the ‘scale’ command to improve model convergence. In addition, sleep staging data from scored polysomnographic recordings were assessed using CRAN R versions 4.3.3 and stats package [46], along with the lmerTest package [48], lme4 [49], gtools version 3.9.5 [52] data.table version 1.16.0, dplyr [53], tidyverse version 2.0.0 [51], MplusAutomation version 1.1.1 [55], lavaan version 0.6–18 [56], psych package version 2.4.6.26 [50], and stats package version 4_4..3.3. Specifically, MANOVA tests were performed to test for differences among condition groups in the percentage of time spent in Rapid Eye Movement (REM) sleep and in Non-Rapid Eye Movement (NREM) sleep stages 1, 2, and 3 during the baseline and first two recovery nights.

In addition to phenotypic analyses, transcriptomic sequences were analyzed. De-multiplexed fastq.gz raw RNA sequence files underwent quality checks using FastQC v0.12.1 [57] and multiqc v.1.14 [58], and mapping against the T2T-CHM13v2.0 reference genome (https://www.ncbi.nlm.nih.gov/datasets/genome/GCF_009914755.1) [59] to generate expression counts at the gene level, with cloud pipeline execution by the Department of Transportation – Secure Data Commons using Amazon Linux platforms. This pipeline involved the use of CutAdapt v4.3 for the removal of the Illumina TruSeq adapters from raw reads, discarding sequence reads shorter than 50 bases, and trimming low-quality bases with the flag –nextseq-trim = 20 [60]. Trimming was followed by paired-read alignment using STAR v.2.7.10b with read length set to 150 bases during the generation of genome indices, and the –outMultimapperOrder Random flag for random output of multimapping reads [61]. Subsequently, featureCounts v2.0.5 was used for strand-specific paired-read generation of expression counts [62]. Chimeric fragments aligned to different chromosomes were discarded by setting the -C flag, and the -d 50 flag was used to reinforce 50 bases as a minimum read length.

Gene expression models largely used default settings, with exceptions specified below, using the limma v. 3.60.4 package of CRAN R v. 4.4.1. Genes with low expression were filtered out (i.e., models only analyzed genes that had at least 1 count per million in as many samples as there were participants), followed by trimmed mean of M values normalization [63]. Linear modeling of each gene was conducted with the voom approach [64], specifically using the function voomLmFit and the Benjamini and Hochberg method to generate False Discovery Rate (FDR) adjusted P-values for multiple testing. Genes were identified as differentially expressed if models yielded an FDR < 0.05 for the factor of interest.

In all gene expression linear models, participant was encoded as a random effect by specifying participant as a blocking variable, and all other terms were additive fixed effects. The cumulative number of hours since midnight (RCH) at the outset of baseline study day 3 (without centering or scaling) was encoded as a numeric covariate in an effort to account for the potential effects of increasing duration at the inpatient facility and repeat test administration. Based on principal component plots suggesting impacts of biological sex and RNA extraction method, these elements were incorporated in models with binary factor terms (male or female, FAA or Baylor). Models were run once on all 59 participants with a factor term to differentiate study groups (CT, DR, NR, TD), and again separately on each condition group with a dataset limited to the 14–16 participants in the group. In each model the final term of interest was either hours of wakefulness or a single neurobehavioral performance metric from the 45-min test battery encoded as a covariate (e.g., PVT lapses). Hours of wakefulness was defined as a count of the total number of scheduled hours of wakefulness since the last TIB sleep episode ended (e.g., for an assay at noon (RCH), if the participant’s most recent TIB ended at 08:00 RCH it was considered 4 h of wakefulness). Each neurobehavioral performance metric (and hours of wakefulness) was modeled separately. Running separate models on each neurobehavioral performance metric of interest on all 59 participants and again separately on each of the four condition groups could be criticized as a form of multiple testing. However, the approach was taken in this exploratory study to allow comparisons of genes differentially expressed relative to the neurobehavioral performance factor of interest in all versus just one sleep condition (CT, DR, NR, or TD) and to maximize the possibility of discovering biomarker candidates.

Finally, gene lists were submitted to QIAGEN Ingenuity Pathway Analysis for a Core Analysis – Expression Analysis [65] to explore molecular pathways and functions. Log2 fold-change values from limma models were used as the data type expression log ratio in Ingenuity Pathway Analysis and selected as the measurement type for Core Analysis runs. All genes passing the low-expression cutoff in limma (≥ 1 count per million in as many samples as participants) were used as the background reference set, and an FDR < 0.05 for the neurobehavioral factor of interest (e.g., PVT lapses) was selected as the filter cutoff to identify the foreground differentially expressed list. Settings were left at default, except for limiting species to mammals and excluding endogenous chemicals from interaction networks.

## Results

From an initial 37,933 inquiries, 73 participants were admitted to the 10-day inpatient study, and after attrition a total of 59 completed the study (Additional file 3: Table S2).

### Sleep staging

**Fig. 2:**
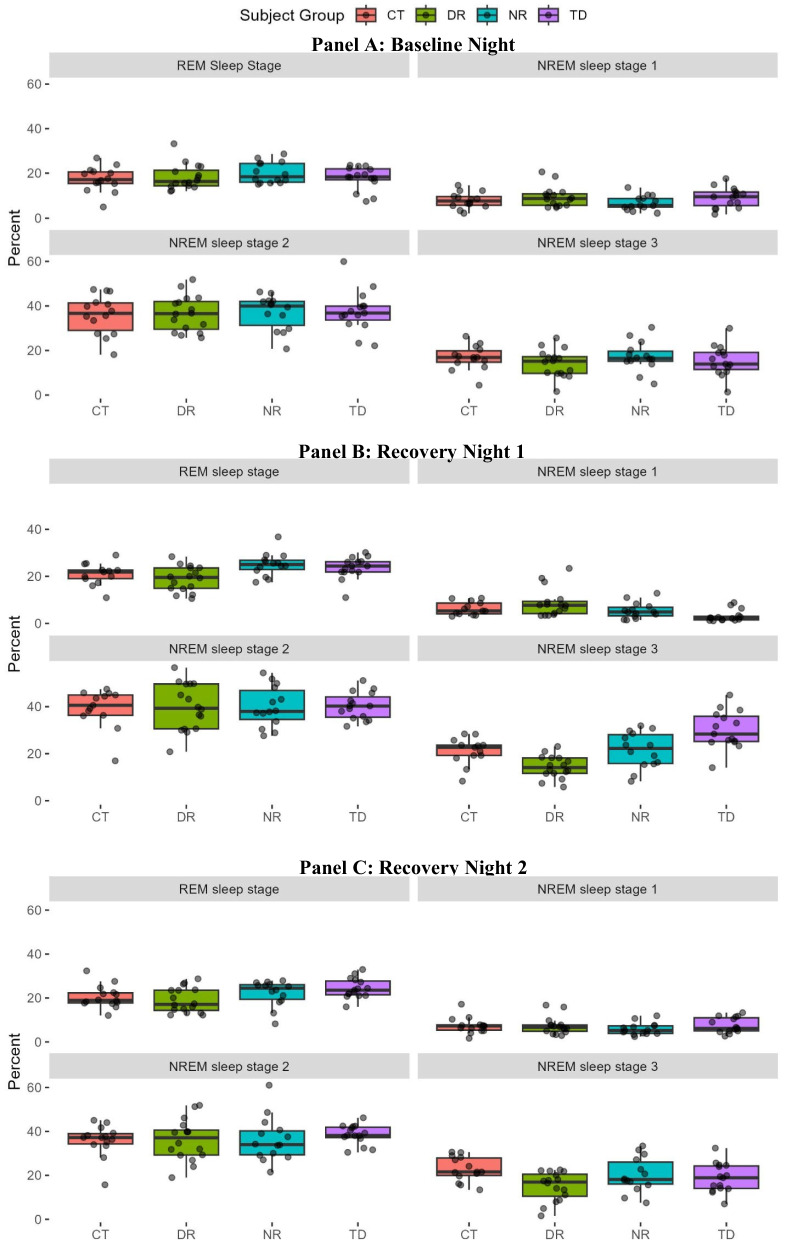
Boxplots of scored polysomnography results of percent of time spent in Non-Rapid Eye Movement (NREM) sleep stages 1, 2, and 3, as well as time in Rapid Eye Movement (REM) sleep. Individual panels show the baseline night before (**A**) and two recovery nights after (**B**, **C**) the experimental segment of altered sleep schedule (e.g., sleep loss and/or altered timing)

There was no significant effect of the study condition group on the percentage of time in Rapid Eye Movement (REM) sleep or in Non-Rapid Eye Movement (NREM) sleep stages 1, 2, or 3 during the baseline night (MANOVA, Wilks *P*-value = 0.83) (Additional file 4: Table S3, Fig. 2). After the experimental segment, there was a significant difference by group during the first night of recovery sleep (*P* = 5.47e-07), with subsequent univariate ANOVA results showing significant differences for NREM sleep stage 1 (*P* = 0.003), NREM sleep stage 3 (*P* = 3.62e-07), and REM sleep (*P* = 0.006), but not NREM sleep stage 2 (*P* = 0.99) sleep. Tukey’s post hoc tests of the first recovery night did not yield significant differences between any of the treatment groups (DR, NR, TD) and the control (CT) group for either NREM sleep stage 1 or REM sleep. Tukey’s tests of NREM sleep stage 3 revealed a significant decrease in the DR relative to CT (*P* = 0.039) and an increase in TD relative to CT (*P* = 0.0037), but no significant difference in the NR from CT (*P* = 0.999). For the second recovery night, the study group yielded an overall effect (Wilks *P*-value = 0.016), but only the NREM sleep stage 3 (*P* = 0.019) and REM sleep (*P* = 0.039) had significant study group effects in univariate ANOVA analyses. Tukey’s post hoc tests on the second recovery night only showed a significant difference from the CT group in the NREM sleep stage 3 analysis of DR from CT (*P* = 0.012), with less time in NREM sleep stage 3 by DR relative to CT participants.

**Fig. 3:**
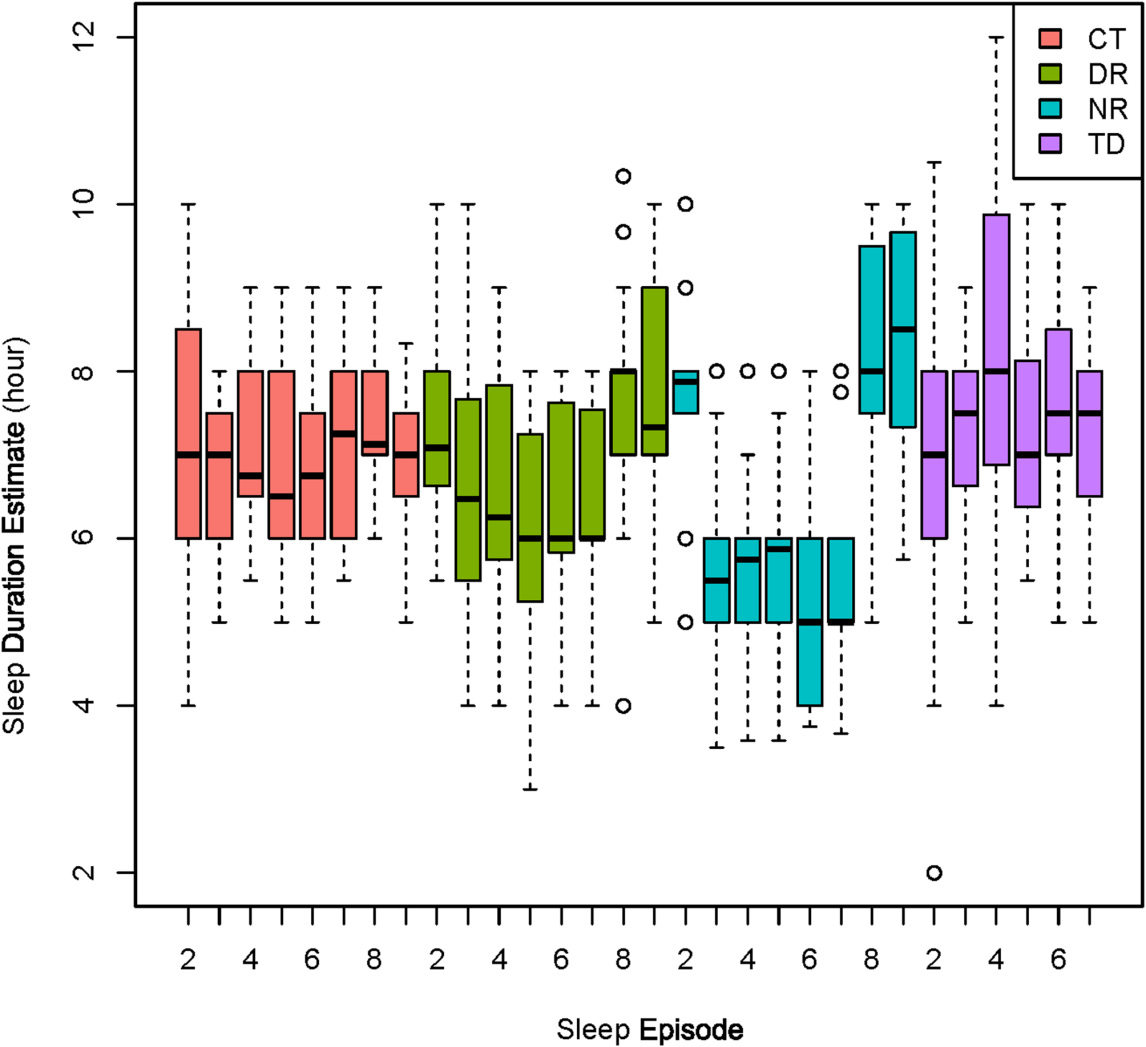
Self-reported inpatient sleep duration estimates for each sleep episode opportunity. For TD participants who lost two nights of sleep during the experimental segment, there are only 7 sleep opportunities. For the others there are 9 sleep episodes, starting with the baseline night (sleep episode 2). Participants in DR and NR had the same total Time In Bed during each sleep episode, with daytime (DR) or nighttime (NR) sleep episodes, despite apparent differences in their self-reported sleep estimates

Both the study condition group and the sleep episode significantly influenced the self-reported estimate of sleep duration. Linear mixed models were generated in lme4 package version 1.1–35.5 with participant as a random effect. Sequentially adding terms with Chi-square model comparisons indicated significant effects of the study condition group (*P* = 0.008) and study sleep episode (*P* = 5.73E-12) on the self-estimated sleep duration. The NR group reported a decrease in their estimated sleep duration from baseline to experimental inpatient sleep episodes on nights 3–7, as expected due to the decrease in TIB from 8 to 5 h during the experimental segment. During this experimental segment, the DR group self-report estimates often exceeded their 5 h of actual TIB (Fig. 3).

### Neurobehavioral performance tests

**Fig. 4:**
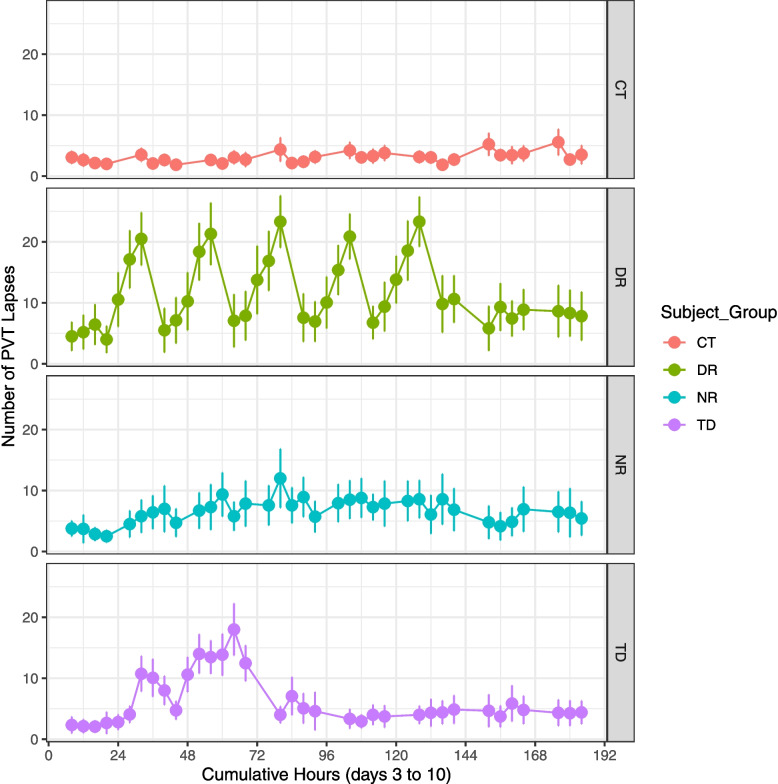
Psychomotor Vigilance Test (PVT) lapses throughout the study. Number (mean ± 1 standard error) of PVT lapses greater than 0.5 s, from midnight (RCH) between study days 2 and 3 until discharge on study day 10 every 4 h during scheduled wake. Data are shown for each of the four study condition groups: control (CT), daytime sleep restriction (DR), nighttime sleep restriction (NR), and total sleep deprivation (TD)

Generalized linear models with an intercept, random term for the participant, fixed term for the study condition group with CT as the reference, and fixed term for the cumulative number of hours into the study yielded significance for the condition group in relation to several neurobehavioral performance test battery metrics (Additional file 5: Table S4). Neurobehavioral performance test metrics that yielded a significant (*P* < 0.05) effect for the DR study group encompassed PVT (with lapses, the square root transform of lapses, or the mean reciprocal reaction time as an endpoint), the overall number of correct responses in the STROOP test (based on either all four colors or just blue-yellow tests), and the two KSS tests conducted at both the start and end of the neurobehavioral battery (Fig. 4, Additional file 2: Figures S2, S3). Only the two KSS tests resulted in *P* < 0.05 for the NR group. Results indicated the TD group experienced the greatest impact of the study protocol on their neurobehavioral performance, with significant impacts on PVT metrics (*P* < 0.05 for lapses or the square root transform of lapses as an endpoint but not the mean reciprocal reaction time), VAS responses for both groggy-clearheaded and alert-sleepy, the overall number correct for blue-yellow tests of STROOP and several metrics based on tests of all four colors and just blue-yellow colors (reaction time for neutral, inhibited, and facilitated tests), the initial and final self-ratings of performance on the PEERS test, initial and final KSS, the results of the FACE assay for both percentage and number correct, the number correct on the DSST, and the adjusted response time for mirror trials in the CVS test (i.e., the average reaction time of mirror trials divided by the number of correct mirror trials). In summary, the KSS was significantly related to the DR, NR, and TD study conditions, but other tests only were significantly related to one (e.g., certain PEERS and VAS responses) or none (e.g., TRACK, MRT, and BART) of the study condition groups.

### Differential gene expression

A total of 1,619 PAXgene blood draws were attempted from participants who completed the 10-day inpatient study (15 TD participants with 27 timepoints, 14 CT with 31 timepoints, 14 NR with 26 timepoints, and 16 DR with 26 timepoints). After the removal of timepoints with failed sample or data collection and those with too few sequence reads per sample (see Methods), a total of 1,572 successfully sequenced RNA samples were used for differential expression analyses across all 59 participants. Raw counts of mapped reads per library (mean ± 1 standard error) were 37.5 million (± 0.3 million).

Thousands of genes were differentially expressed relative to hours of wakefulness. When replacing the hours of wakefulness term with a metric from the 45-min neurobehavioral performance test battery, the number of differentially expressed genes (FDR < 0.05) varied widely depending on the specific test metric and the study condition group(s) analyzed (Additional file 6: Table S5; Additional file 7–11: Table S6). For some tests there were no differentially expressed genes in any study group (e.g., BART metrics, percent correct in DSST, and the number correct in MRT beginning and intermediate level trials). For the STROOP test, the number of differentially expressed genes varied widely depending on the specific STROOP test endpoint. For PVT there was differential gene expression in tests of all and each individual study group for PVT lapses, transformed lapses, and the mean reciprocal reaction time, except for the PVT lapses metric for the CT and NR groups.

From each of the 10 neurobehavioral performance test categories (e.g., PVT), one metric was selected (e.g., number of PVT lapses) for comparison of the number of differentially expressed genes (Additional file 12: Table S7A). Based on this, a total of 39 genes were differentially expressed (FDR < 0.05) in at least half of the 10 neurobehavioral performance tests for one or more of the study group comparisons (ALL, CT, DR, NR, TD) (Additional file 12: Tables S7B and S7C). For the analysis of all 59 participants, a handful of genes were differentially expressed in at least 5 of the 10 tests: *A-Kinase Anchoring Protein 5* (*AKAP5*), *Epiregulin* (*EREG*), *Golgi Associated Kinase 1B* (*GASK1B*), and *Ubiquitin Conjugating Enzyme E2 J1* (*UBE2J1*) (Figures S4-S7). Of these, *AKAP5* and *EREG* were significantly related to five of the neurobehavioral tests in DR participants, and *UBE2J1* was significant in six of the tests for TD participants (Additional file 12: Tables S7C). For any of the participant groups (ALL, CT, DR, NR, or TD), 89% or more of the instances of differential expression represented cases where a gene was significant for only one or two of the neurobehavioral performance test metrics.

*STEAP4 metalloreductase* (*STEAP4*) was among the genes differentially expressed relative to PVT lapses in the TD group. This gene also was shown to be associated with PVT lapses during total sleep deprivation in two prior studies: a total RNA sequencing study in collaboration with the Naval Medical Research Unit-Dayton (NAMRU-D, placebo 8 timepoint study run) [26], and a microarray analysis in collaboration with Washington State University (WSU) [27] (Additional file 2: Figure S8). Other differentially expressed genes reported in the present study that also were related to PVT lapses in the WSU report encompassed *EFHD2*, *AQP9*, *LITAF*, *MSL1*, *CXCR1*, *KCNJ15*, *FLOT1*, and *ELOVL5*. Altogether 59 genes were related to PVT lapses in the TD group of this study and the NAMRU-D study (placebo 8 timepoint study run) (Additional file 13: Table S8). To further the comparison, a similar modeling approach to that used in the NAMRU-D study was applied to the present TD group dataset, with use of the R package edgeR version 4.2.1 to generate generalized linear models of the counts data with a negative binomial distribution without log transformation (rather than the present study’s linear modeling approach). For these models, fixed effects were incorporated for the individual participant, PVT lapses, RNA extraction (by FAA or Baylor), and two terms to capture cyclical rhythms: cyc1 = sin(2 * pi * hour/24), and cyc2 = cos(2 * pi * hour/24), where hour was 0 (midnight), 4 (04:00), 8 (08:00), 12 (noon), 16 (16:00), or 20 (20:00) (RCH) based on timing of the blood draw. This resulted in a decrease from 59 to 39 differentially expressed genes related to PVT lapses during total sleep deprivation detected in both the NAMRU-D and the present study, of which all but two also were differentially expressed in the present study with the original linear modeling approach. A few of those genes were significantly related to at least five of the neurobehavioral tests in the original models of the TD group for the present study: *Acyl-CoA Synthetase Long Chain Family Member 1* (*ACSL1*), *CDC42 Effector Protein 3* (*CDC42EP3*) (Additional file 2: Figure S12), *MIR3945 Host Gene* (*MIR3945HG*), *Phospholipase B Domain Containing 1* (*PLBD1*), and *UBE2J1*. Based on the original models for the present study, the gene *STEAP4* was significantly differentially expressed relative to not only PVT (TD group only, endpoints of mean reciprocal reaction time, lapses, and square root transformed lapses), but also to hours of wakefulness (ALL, CT, and DR comparisons), to the initial KSS (ALL, DR, and NR) and final KSS (DR), the initial PEERS ratings of effort and performance (NR group), the TRACK losses (NR group), and VAS ratings of sleepy-alert and of groggy-clearheadedness (ALL, DR, NR).

### Functional significance and pathways

**Fig. 5:**
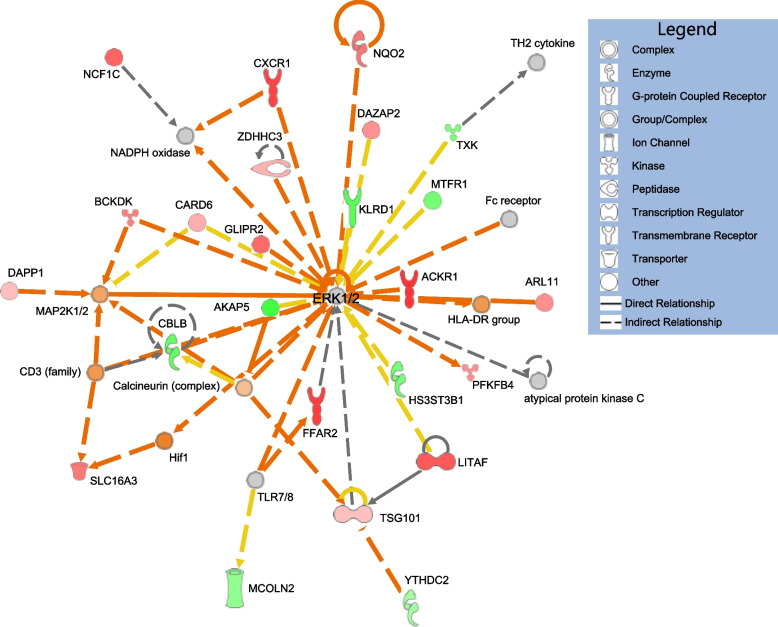
Predicted molecular pathway related to Psychomotor Vigilance Test (PVT) impairment. Network diagram predicted with QIAGEN Ingenuity Pathway Analysis from a core analysis of genes differentially expressed relative to PVT lapses from participants in the Total Sleep Deprivation (TD) group. Orange lines indicate relationships between molecules leading to activation; yellow lines indicate relationships are inconsistent with the downstream state of the molecule; gray lines indicate effect not predicted. Green molecules show decreased expression measurement, red show increased expression, orange indicate predicted activation, and gray indicate effect not predicted

Based on the expected functions of molecules and their interactions from literature, Ingenuity Pathway Analysis interpretations of genes differentially expressed relative to neurobehavioral performance test scores revealed functions related to inflammation and the immune system. For genes differentially expressed relative to PVT lapses in the TD participant group, two of the top canonical pathways were neutrophil degranulation (P-value 4.46E-17) and pathogen-induced cytokine storm signaling pathway (P-value 9.26E-09). Many of the upstream regulators based on an upstream analysis were cytokines, such as Tumor Necrosis Factor (TNF) (P-value 1.49E-08). In the DR group, which was associated with far fewer differentially expressed genes for PVT lapses, TNF still appeared as an upstream regulator (P-value 4.27E-02). Among the many networks predicted for the TD group based on genes related to PVT lapses was one centered on Extracellular Signal-Related Kinases 1/2 (ERK1/2), and involved differentially expressed genes *AKAP5*, *CXCR1*, and *LITAF* as well as predicated activation of the Hypoxia Inducible Factor 1 (HIF1) complex containing members *Aryl Hydrocarbon Receptor Nuclear Translocator* (*ARNT*) and *Hypoxia Inducible Factor 1 Subunit Alpha* (*HIF1A*) (Fig. 5). This network has associations with cellular assembly and organization, cellular movement, and skeletal and muscular system development and function. Another network with functions in cellular movement, lipid metabolism, and small molecule biochemistry centered on the AKT family, consisting of members *AK1*, *AKT2*, and *AKT3*.

## Discussion

This study expands current understanding of the human response to sleep loss and altered timing of sleep, demonstrating effects on PSG-scored sleep stages, subjective assessments of sleep, neurobehavioral performance, and molecular gene expression. In addition to identifying reproducible biomarker candidates related to attention impairment (i.e., PVT lapses) during total sleep deprivation, the current report expands the candidate pool to genes associated with impairment on multiple neurobehavioral performance tests. The simulated shiftwork group (DR) with 5 h of daytime sleep opportunities across 5 days had scores on certain neurobehavioral performance tests such as the PVT of similar or greater magnitude than the acute total sleep deprivation (TD) group, a group that experienced two nights of total sleep loss (Fig. 4, Additional file 5: Table S4). A caveat to attributing such findings to sleeping during the day and circadian disruption is that the DR group began the experimental segment with both decreased quantity and altered timing of sleep in order to accommodate the experimental intervention schedule. Participants were kept awake from 06:45am (RCH) on baseline day 3 until their next Time In Bed opportunity starting at 09:45am (RCH) on study day 4, a duration of 27 h. Some night shiftworkers may choose to rest (e.g., nap) prior to beginning night shiftwork, which would decrease their sleep loss. Yet in the present study, the intervention schedule of the DR group mimics what may occur for a night shiftworker transitioning from nighttime sleep on a day off to daytime sleep following the first night shift. The protocol reflects sleep loss associated with the first night shift of a sequence, which may be a common experience for night shiftworkers [66, 67].

While average neurobehavorial test performance in the DR group often returned to baseline levels after the short 5-h TIB opportunity during the experimental segment, it showed substantial deviations during subsequent time awake likely reflecting both wake-dependent and circadian impacts (Fig. 4). Polysomnographic recordings and subjective self-assessments indicated an effect of both TD and DR study conditions on sleep stages. As reviewed in [68], an increase in slow-wave sleep is expected during the rebound or recovery sleep following sleep deprivation. This is congruent with the current study PSG recordings, in which the TD group showed a decrease in NREM sleep stage 1 and an increase in NREM sleep stage 3 on the first recovery night (Fig. 2, Additional file 4: Table S3). Effects for the DR group continued into the second recovery night. Whereas DR participants received the same sleep opportunity during the experimental segment as the NR group, the DR group notably subjectively overestimated their time asleep during these 5 days (Fig. 3), with average values typically exceeding 6 h. In contrast, the NR group generally reported estimates closer to their actual allowed 5-h TIB (Fig. 3). Notwithstanding the study’s limitations, these findings are consistent with the potential for altered sleep timing and circadian disruption during shiftwork to both alter physiology and impair self-assessment [69].

Overall, results suggest the TD group generally exhibited the most impairment on neurobehavioral performance tests (Additional file 5: Table S4), but impacts varied among specific tests with substantial impacts also seen for DR participants. For example, the mean number of overall correct responses on STROOP assays (blue-yellow tests only) dropped to the lowest rates for the DR group (Additional file 2: Figure S3). As previous studies have shown [70], observed impacts of sleep loss or altered timing may differ depending which aspect of neurobehavioral performance is investigated. During the first few neurobehavioral assessments after awakening, participants in the DR group self-reported sleepiness on the KSS at near-baseline levels. They subsequently showed dramatic increases in self-rated sleepiness, with their pre-sleep 07:15–08:00 (RCH) neurobehavioral assessments on average rising to values near the peak for the TD group during acute total sleep deprivation (Additional file 2: Figure S2). A review of more objective metrics, such as PVT lapses (Fig. 4), also suggests that the simulated shiftwork DR group may experience neurobehavioral performance deficits comparable to acute total sleep deprivation.

Differentially expressed genes associated with neurobehavioral performance impairment varied with the specific neurobehavioral performance metric and also depending on the participant condition group (Additional file 6: Table S5; Additional file 7–11: Table S6): all 59 participants (ALL), control (CT), daytime restricted sleep (DR), nighttime restricted sleep (NR), and total sleep deprivation (TD). No biomarker candidates were found associated with risk-taking behavior as measured with the BART test. However, there were 22 differentially expressed genes relative to PVT lapses in tests of all participants, 73 for the DR group, and 952 for the TD group. For spatial orientation as probed with the TRACK test, by far the greatest number of biomarker candidates occurred for the NR group, with 2,085 differentially expressed genes. A small fraction of genes such as *AKAP5* were differentially expressed relative to multiple neurobehavioral test metrics and participant groups (Additional file 12: Table S7), which indicates the potential for developing a generic panel of biomarker genes to detect an overall state of fatigue-related impairment. While such genes could be pleiotropic, a mechanistic understanding and an evaluation of whether the expression patterns represent causal vs. correlative relationships between molecular changes and fatigue-related neurobehavioral performance requires further research. Most genes were uniquely differentially expressed relative to specific neurobehavioral test metrics or participant subsets. Hence if a more tailored approach is desired for detecting specific forms of fatigue-related impairment, in recognition that different occupations or aspects of well-being may warrant closer monitoring of distinct aspects of neurobehavioral performance, such tailoring may be possible using the genes that only respond to certain conditions or neurobehavioral performance metrics. For example, the *PLBD1* gene may be a better indicator of acute sleep loss than altered sleep timing or restriction, given its detection in multiple neurobehavioral performance metrics within TD but not DR or NR participants. Expression of *PLBD1* also may be a possible biomarker for left ventricular dysfunction [71].

In general, the relation between gene expression and fatigue-related neurobehavioral performance impairment appears complex. Many genes are known to be rhythmic in their expression and may even serve as indicators of circadian phase [72, 73]. In the present study, some of the genes differentially expressed relative to neurobehavioral impairment metrics also appeared to exhibit a rhythmic pattern of expression in baseline conditions, that upon visual inspection may be altered in the sleep intervention groups DR, NR, and TD (Additional file 2: Figures S5, S7, S8, S9). This supports prior work in which a clustering analysis of genes related to PVT lapses showed a profile congruent with circadian rhythm disruption during an experimental segment of total sleep deprivation [27]. Such findings could reflect a mechanism whereby sleep loss or altered sleep timing causes altered gene expression, and detection of the genes as related to neurobehavioral impairment is due to independent, concurrent effects of sleep loss on gene expression and on the impairment metric. Causal biomarkers would be invaluable for advancing mechanistic insights, but for the purpose of developing surrogate metrics for fatigue-related neurobehavioral performance impairment, a panel of strongly correlated biomarkers may suffice.

Understanding of causal vs. correlative relationships is beyond the scope of the current study, but functional roles of the genes identified as differentially expressed does support validity of the findings and may lend to development of hypotheses for future studies. Core analyses run in Ingenuity Pathway Analysis of genes differentially expressed relative to PVT lapses in the TD group resulted in a predicted network centered on the *Extracellular Signal Related Kinase* (*ERK*) genes (Fig. 5), which functions as reviewed in [74] as sleep-promoting kinases. Core analyses of PVT-associated genes in both TD and DR participant groups indicated upstream regulatory roles for Tumor Necrosis Factor (TNF), congruent with literature suggesting a role of this molecule and more generally inflammation and the immune response in cognitive deficits and sleep regulation [75, 76]. There is reported evidence that a sequence variant in the gene *TNFa* is associated with inherited resistance to PVT impairment during total sleep deprivation [25]. Another molecule of particular interest is the gene *STEAP4*, which was found in this study and two prior works in association with PVT lapses during total sleep deprivation [26, 27]. This gene, also known as *STAMP2*, has been implicated in the inflammatory response and atherosclerosis [77]. Potentially, *STEAP4* could be involved in the previously identified association between insufficient sleep and increased risk of cardiovascular disease [2–5]. Furthermore, in the present study the gene *AKAP5* was associated with multiple neurobehavioral performance test metrics. This gene is transcribed and translated to generate a scaffolding protein, with roles as a signal integrator and impacts on long-term synaptic plasticity [78, 79]. Investigations of such genes and their molecular pathways may yield pharmacological targets for developing countermeasures to the effects of sleep loss, or new understanding of ways in which sleep loss impacts health and cognition.

There are multiple limitations to this study, such as the small number (14–16) of participants per study condition group. Melatonin data were not available, and thus this report does not explicitly model or address circadian rhythms, which are known to affect sleep, neurobehavioral metrics, and gene expression. While these limitations reduce the ability to interpret findings and attribute mechanisms, the study nonetheless presents several associations of neurobehavioral and molecular changes that can be explored in future work. Running separate statistical analyses for gene expression related to multiple neurobehavioral performance test outcomes on all 59 participants, and again separately on each of the four study condition groups, may risk false positive results from multiple testing. The risk was accepted for this biomarker study because the aim was to identify potential genes of interest for further validation work, and thus false positives were deemed more acceptable than missing candidate biomarkers. All genes with FDR < 0.05 for one or more neurobehavioral performance tests, and particularly the objective tests rather than KSS, PEERS, and VAS scales, could be considered candidate biomarkers of fatigue-related neurobehavioral performance impairment. Further work is needed to validate the findings.

Power analyses were not conducted. Large inter-individual variation in responses to sleep loss and the limited amount of published data relating neurobehavioral performance impairment to gene expression during sleep loss, meant that effect sizes for key metrics would be difficult to estimate with any reliability. The study population was ages 20–45 years. This was intended to increase application of findings to young and middle-aged working adults, but may add noise by adding variability due to well-known changes in sleep and neurobehavioral performance across the lifespan, as recently reviewed by Simon et al. [80]. Results should be considered exploratory; it is the authors’ hope that the considerable time series dataset and trends presented will stimulate further research.

## Conclusion

The current study identifies biomarker candidate genes for monitoring neurobehavioral performance impairment from reduced quantity or shifted timing of sleep. Some genes such as *STEAP4* were previously identified in association with PVT lapses during total sleep deprivation, suggesting the biomarker findings are reproducible. The discovery of *AKAP5* gene expression changes in relation to multiple neurobehavioral performance metrics may be of particular interest. The gene has a significant role in memory-related brain circuits, and has been proposed as a target for precision medicine and pharmacology towards treatment of neurological illnesses [81]. The present study advances prior work by identifying new candidates across multiple assays of neurobehavioral performance, and extends the type of sleep loss from acute total sleep deprivation to also incorporate sleep restriction and altered timing of sleep (daytime versus nighttime sleep). Self-reported estimates of sleep timing, sleep staging with polysomnography data, neurobehavioral performance assessments, and transcriptomics all indicate effects of both reducing quantity and altering timing of sleep. Altogether these data provide a more comprehensive understanding of the impacts of sleep loss. Molecular assessments may one day be a useful tool to augment impairment monitoring and risk management practices, and may be particularly useful in cases such as postmortem accident investigation as a novel approach to infer potential fatigue-related impairment. In addition to furthering the goal of identifying candidate molecular indicators for fatigue-related neurobehavioral performance impairment, the study provides a wealth of data for future investigations. Indeed, the thousands of timepoints of physiological and neurobehavioral data collected may augment efforts to better understand the ramifications of adequate sleep for safety and well-being. Next steps could involve the use of advanced big data analytics and machine learning to aid biomarker and sleep health discoveries.

## Supplementary Information

Additional file 1: Table S1A. Schedule for CT participants, indicating TIB sleep opportunities, 45-minute periods for neurobehavioral performance test batteries, meals, and whole blood collection into PAXgene® RNA blood tubes. Practice assays and ad lib meals on acclimation days 1-2 are not shown. Times are Relative Clock Hours. Table S1B. Schedule for DR participants, indicating TIB sleep opportunities, 45-minute periods for neurobehavioral performance test batteries, meals, and whole blood collection into PAXgene® RNA blood tubes. Practice assays and ad lib meals on acclimation days 1-2 are not shown. Times are Relative Clock Hours. Table S1C. Schedule for NR participants, indicating TIB sleep opportunities, 45-minute periods for neurobehavioral performance test batteries, meals, and whole blood collection into PAXgene® RNA blood tubes. Practice assays and ad lib meals on acclimation days 1-2 are not shown. Times are Relative Clock Hours. Table S1D. Schedule for TD participants, indicating TIB sleep opportunities, 45-minute periods for neurobehavioral performance test batteries, meals, and whole blood collection into PAXgene® RNA blood tubes. Practice assays and ad lib meals on acclimation days 1-2 are not shown. Times are Relative Clock Hours

Additional file 2: Figure S1. CONSORT diagram of participant recruitment, ending with the number of participants that completed each of the four study condition groups: control (CT), daytime sleep restriction (DR), nighttime sleep restriction (NR), and total sleep deprivation (TD). Initially potential participants were directed to an online questionnaire, followed by participant online and in-person based screening procedures. Participants who passed screening criteria were admitted to the inpatient study. Figure S2. Mean (+/- 1 standard error) of Karolinska Sleepiness Scale scores from midnight at the outset of baseline study day 3 through the end of the 10-day inpatient study, reflecting the initial Karolinska Sleepiness Scale towards the beginning of each ~45-minute neurobehavioral test battery. All times are Relative Clock Hour (RCH). Figure S3. Mean (+/- 1 standard error) of the number of correct responses on blue-yellow tests only for the STROOP results. Scores are shown from midnight at the outset of baseline study day 3 through the end of the 10-day inpatient study. All times are Relative Clock Hour (RCH). Figure S4. Mean (+/- 1 standard error) log_2_ counts per million gene expression, based on normalized libraries, for the gene *AKAP5*. All times are Relative Clock Hour (RCH), showing study day 3 through the end of the 10-day inpatient study. Figure S5. Mean (+/- 1 standard error) log_2_ counts per million gene expression, based on normalized libraries, for the gene *EREG*. All times are Relative Clock Hour (RCH), showing study day 3 through the end of the 10-day inpatient study. Figure S6. Mean (+/- 1 standard error) log_2_ counts per million gene expression, based on normalized libraries, for the gene *GASK1B*. All times are Relative Clock Hour (RCH), showing study day 3 through the end of the 10-day inpatient study. Figure S7. Mean (+/- 1 standard error) log_2_ counts per million gene expression, based on normalized libraries, for the gene *UBE2J1*. All times are Relative Clock Hour (RCH), showing study day 3 through the end of the 10-day inpatient study. Figure S8. Mean (+/- 1 standard error) log_2_ counts per million gene expression, based on normalized libraries, for the gene *STEAP4*. All times are Relative Clock Hour (RCH), showing study day 3 through the end of the 10-day inpatient study. Figure S9. Mean (+/- 1 standard error) log_2_ counts per million gene expression, based on normalized libraries, for the gene *CDC42EP3*. All times are Relative Clock Hour (RCH), showing study day 3 through the end of the 10-day inpatient study.

Additional file 3: Table S2. Demographics and information for participants who completed the 10-day inpatient study. Values for the Apnea Hypopnea Index (AHI) and Periodic Leg Movement (PLM) index were generated from scoring home sleep test results

Additional file 4: Table S3. Scored polysomnography results per sleep episode, based on 30-second epochs. See the worksheet tab Description of Variables for a description of column headers

Additional file 5: Table S4. List of neurobehavioral performance test battery variables and P-values for model terms of the intercept; treatment study condition groups of DR, NR, and TD; and cumulative hours since midnight (RCH) between study days 2-3 (start of baseline) into the 10-day inpatient study. For a description explaining the meaning of variable terms, see worksheet tab Description of Variables. Worksheet tab Model Syntax provides the model family and syntax used to generate results

Additional file 6: Table S5. Number of genes differentially expressed (FDR<0.05) for the neurobehavioral performance test battery noted in the column header. For a description of neurobehavioral performance test battery tests and endpoints, see worksheet tab Description of Variables

Additional file 7: Table S6A. List of FDR values for each gene tested, with respect to the noted neurobehavioral test battery endpoint and participant condition group ALL. If an FDR is not available (e.g., genes with too few counts in a given participant category, an NA is listed. See the Description of Variables for the meaning of column headers (neurobehavioral test battery endpoints). Each row represents a gene (NCBI Gene Symbol). Genes with FDR<0.05 are considered significantly differentially expressed for the neurobehavioral test battery in the column header and participant group

Additional file 8: Table S6B. List of FDR values for each gene tested, with respect to the noted neurobehavioral test battery endpoint and participant condition group CT. If an FDR is not available (e.g., genes with too few counts in a given participant category, an NA is listed. See the Description of Variables for the meaning of column headers (neurobehavioral test battery endpoints). Each row represents a gene (NCBI Gene Symbol). Genes with FDR<0.05 are considered significantly differentially expressed for the neurobehavioral test battery in the column header and participant group

Additional file 9: Table S6C. List of FDR values for each gene tested, with respect to the noted neurobehavioral test battery endpoint and participant condition group DR. If an FDR is not available (e.g., genes with too few counts in a given participant category, an NA is listed. See the Description of Variables for the meaning of column headers (neurobehavioral test battery endpoints). Each row represents a gene (NCBI Gene Symbol). Genes with FDR<0.05 are considered significantly differentially expressed for the neurobehavioral test battery in the column header and participant group

Additional file 10: Table S6D. List of FDR values for each gene tested, with respect to the noted neurobehavioral test battery endpoint and participant condition group NR. If an FDR is not available (e.g., genes with too few counts in a given participant category, an NA is listed. See the Description of Variables for the meaning of column headers (neurobehavioral test battery endpoints). Each row represents a gene (NCBI Gene Symbol). Genes with FDR<0.05 are considered significantly differentially expressed for the neurobehavioral test battery in the column header and participant group

Additional file 11: Table S6E. List of FDR values for each gene tested, with respect to the noted neurobehavioral test battery endpoint and participant condition group TD. If an FDR is not available (e.g., genes with too few counts in a given participant category, an NA is listed. See the Description of Variables for the meaning of column headers (neurobehavioral test battery endpoints). Each row represents a gene (NCBI Gene Symbol). Genes with FDR<0.05 are considered significantly differentially expressed for the neurobehavioral test battery in the column header and participant group

Additional file 12: Table S7A. List of FDR values for each gene relative to one endpoint for each of the distinct neurobehavioral performance tests, for a given participant subset: ALL 59 participants, or those in the control (CT), daytime sleep restriction (DR), nighttime sleep restriction (NR), or total sleep deprivation (TD) group. For a description of neurobehavioral performance test endpoints, see worksheet tab Description of Variables. Table S7B. List of the number of times each tested gene was significantly differentially expressed relative to one endpoint of the distinct neurobehavioral performance tests. Values represent a count of the number of times the gene was significantly differentially expressed (FDR<0.05) for a given participant subset: ALL 59 participants, or those in the control (CT), daytime sleep restriction (DR), nighttime sleep restriction (NR), or total sleep deprivation (TD) group. For a description of neurobehavioral performance test endpoints, see worksheet tab Description of Variables. Table S7C. List of genes significantly differentially expressed relative to at least 5 distinct neurobehavioral performance test assays. Values represent the number of times the gene was counted as significant (FDR<0.05) for a given participant subset: ALL 59 participants, or those in the control (CT), daytime sleep restriction (DR), nighttime sleep restriction (NR), or total sleep deprivation (TD) group. For a description of neurobehavioral performance test endpoints, see worksheet tab Description of Variables

Additional file 13: Table S8. Significance (FDR<0.05) values for gene expression relative to PVT lapses in the NAMRU-D study by Uyhelji et al. 2023, the current study with the original linear modeling approach using limma voom, and the current study re-run with a negative binomial distribution in edgeR similar to the NAMRU-D approach. Only genes tested in all 3 approaches and with a significant value (FDR<0.05) in the NAMRU-D study are shown

## Data Availability

Data analyzed during this study are available in this published article and its supplementary information files, with additional datasets available online at the National Center for Biotechnology Information database of genotypes and phenotypes (dbGaP) at https://www.ncbi.nlm.nih.gov/gap/ under the accession identifier phs003924.v1.p1.
