# Supplementary material for: Exploratory development of biomarkers for neurobehavioral performance impairment during sleep loss: comparison across multiple types of sleep deprivation
Source: BMC Genomics. 2025 Nov 14;26:1043. doi: 10.1186/s12864-025-12193-6 (PMC12619276; doi:10.1186/s12864-025-12193-6)
Supplement: Supplementary file 2 — Additional file 2: Figure S1. CONSORT diagram of participant recruitment, ending with the number of participants that completed each of the four study condition groups: control (CT), daytime sleep restriction (DR), nighttime sleep restriction (NR), and total sleep deprivation (TD). Initially potential participants were directed to an online questionnaire, followed by participant online and in-person based screening procedures. Participants who passed screening criteria were admitted to the inpatient study. Figure S2. Mean (+/- 1 standard error) of Karolinska Sleepiness Scale scores from midnight at the outset of baseline study day 3 through the end of the 10-day inpatient study, reflecting the initial Karolinska Sleepiness Scale towards the beginning of each ~45-minute neurobehavioral test battery. All times are Relative Clock Hour (RCH). Figure S3. Mean (+/- 1 standard error) of the number of correct responses on blue-yellow tests only for the STROOP results. Scores are shown from midnight at the outset of baseline study day 3 through the end of the 10-day inpatient study. All times are Relative Clock Hour (RCH). Figure S4. Mean (+/- 1 standard error) log2 counts per million gene expression, based on normalized libraries, for the gene AKAP5. All times are Relative Clock Hour (RCH), showing study day 3 through the end of the 10-day inpatient study. Figure S5. Mean (+/- 1 standard error) log2 counts per million gene expression, based on normalized libraries, for the gene EREG. All times are Relative Clock Hour (RCH), showing study day 3 through the end of the 10-day inpatient study. Figure S6. Mean (+/- 1 standard error) log2 counts per million gene expression, based on normalized libraries, for the gene GASK1B. All times are Relative Clock Hour (RCH), showing study day 3 through the end of the 10-day inpatient study. Figure S7. Mean (+/- 1 standard error) log2 counts per million gene expression, based on normalized libraries, for the gene UBE2J1. All times are Relat [file 12864_2025_12193_MOESM2_ESM.docx]

**
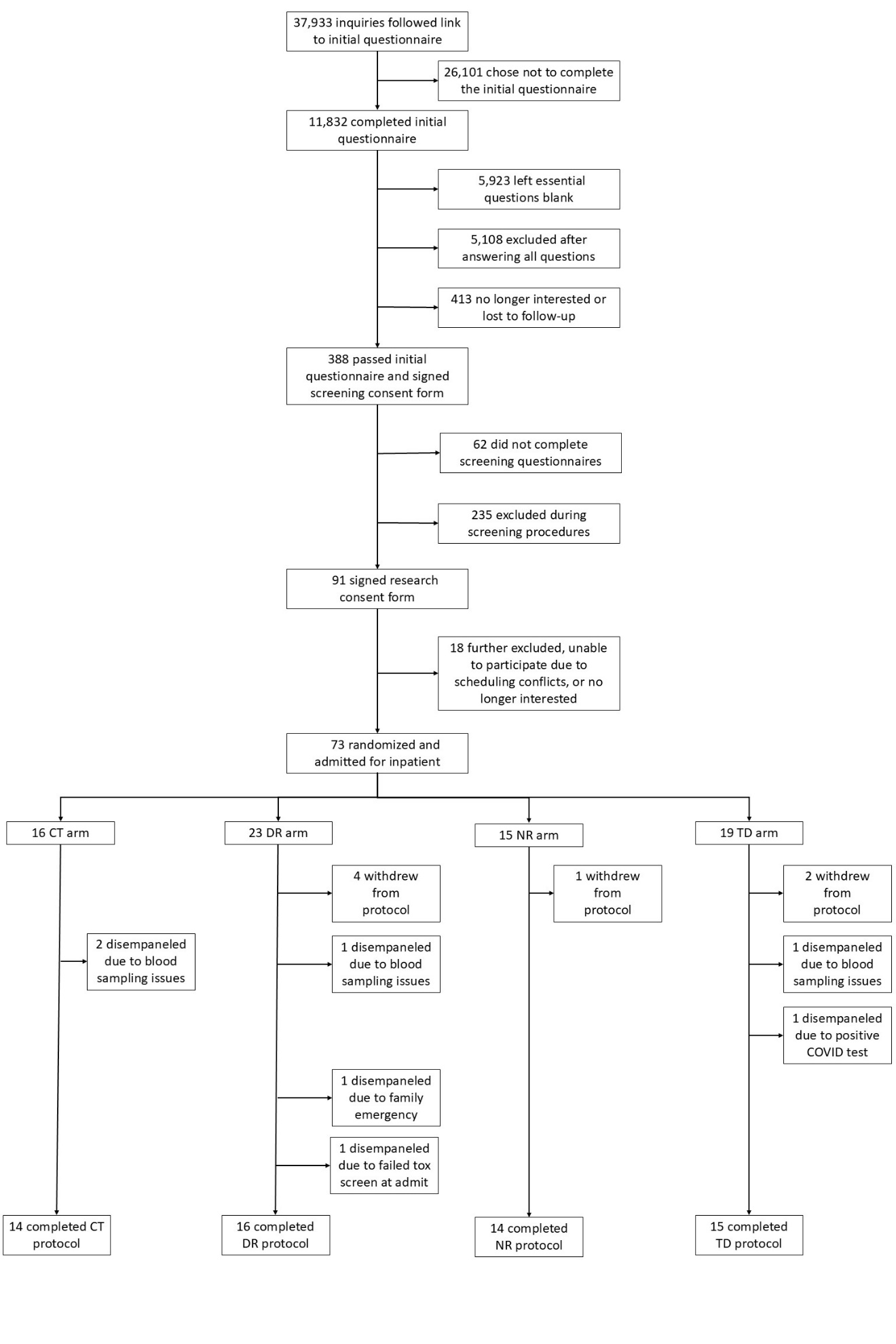
**

**Figure S1**. CONSORT diagram of participant recruitment, ending with the number of participants that completed each of the four study condition groups: control (CT), daytime sleep restriction (DR), nighttime sleep restriction (NR), and total sleep deprivation (TD). Initially potential participants were directed to an online questionnaire, followed by participant online and in-person based screening procedures. Participants who passed screening criteria were admitted to the inpatient study.


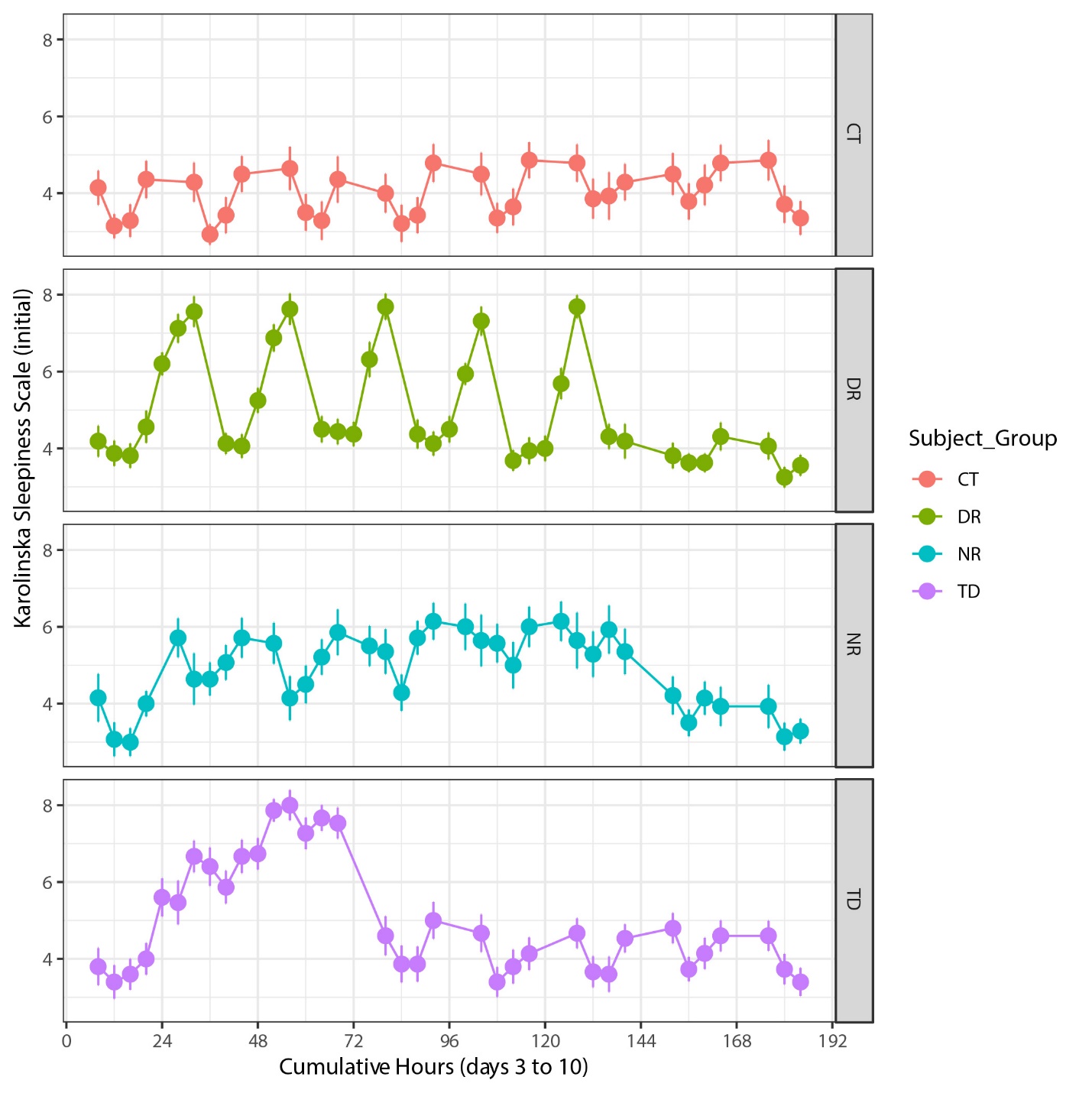


**Figure S2**. Mean (+/- 1 standard error) of Karolinska Sleepiness Scale scores from midnight at the outset of baseline study day 3 through the end of the 10-day inpatient study, reflecting the initial Karolinska Sleepiness Scale towards the beginning of each ~45-minute neurobehavioral test battery. All times are Relative Clock Hour (RCH).


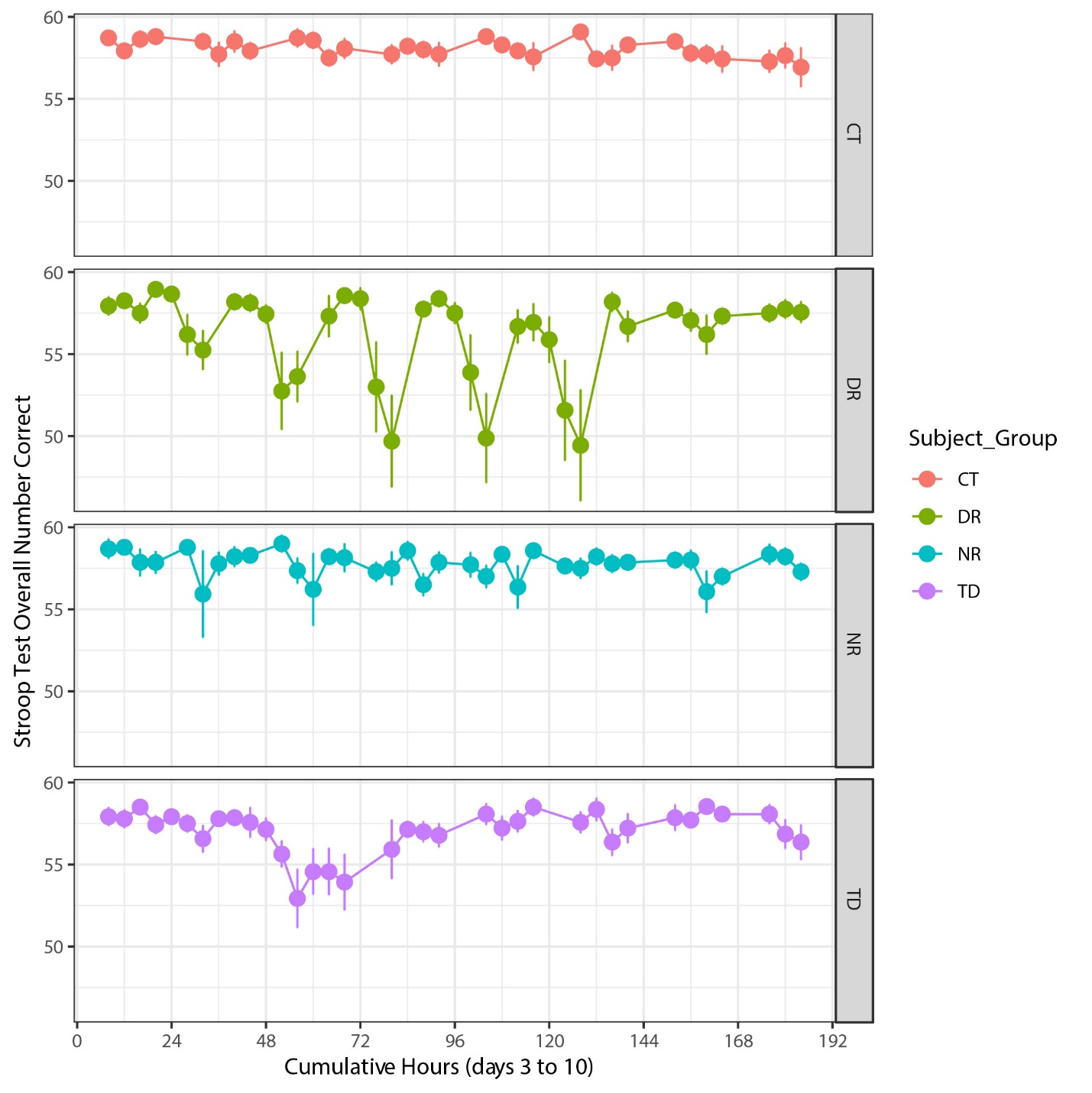


**Figure S3.** Mean (+/- 1 standard error) of the number of correct responses on blue-yellow tests only for the STROOP results. Scores are shown from midnight at the outset of baseline study day 3 through the end of the 10-day inpatient study. All times are Relative Clock Hour (RCH).


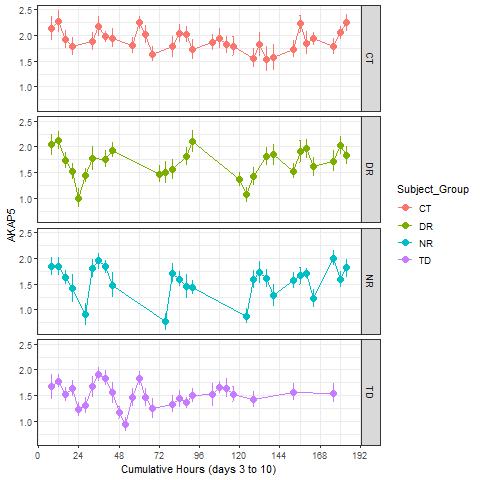


**Figure S4.** Mean (+/- 1 standard error) log2 counts per million gene expression, based on normalized libraries, for the gene *AKAP5*. All times are Relative Clock Hour (RCH), showing study day 3 through the end of the 10-day inpatient study.


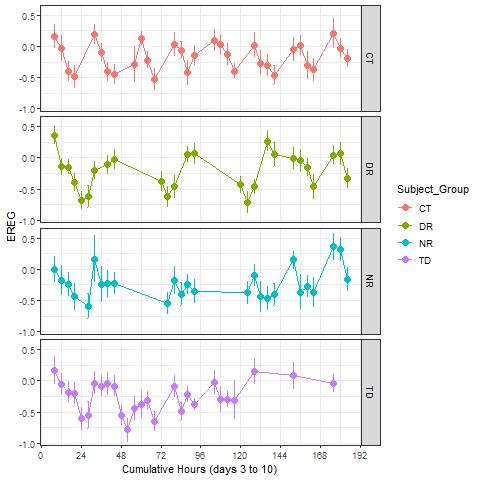


**Figure S5.** Mean (+/- 1 standard error) log2 counts per million gene expression, based on normalized libraries, for the gene *EREG*. All times are Relative Clock Hour (RCH), showing study day 3 through the end of the 10-day inpatient study.


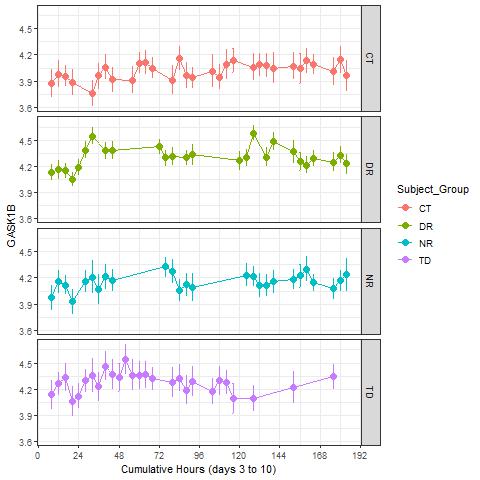


**Figure S6.** Mean (+/- 1 standard error) log2 counts per million gene expression, based on normalized libraries, for the gene *GASK1B*. All times are Relative Clock Hour (RCH), showing study day 3 through the end of the 10-day inpatient study.


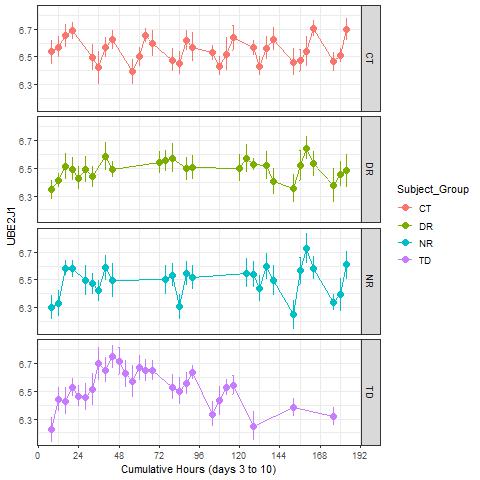


**Figure S7.** Mean (+/- 1 standard error) log2 counts per million gene expression, based on normalized libraries, for the gene *UBE2J1*. All times are Relative Clock Hour (RCH), showing study day 3 through the end of the 10-day inpatient study.


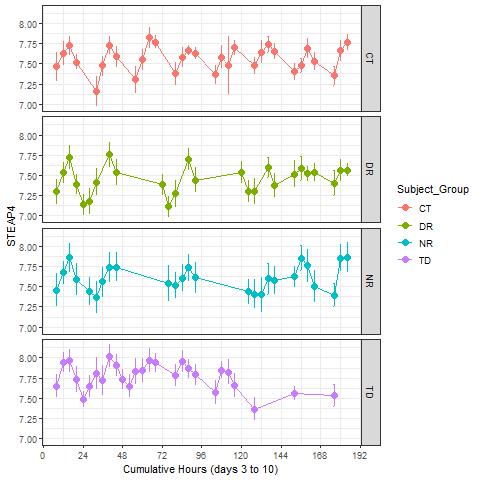


**Figure S8.** Mean (+/- 1 standard error) log2 counts per million gene expression, based on normalized libraries, for the gene *STEAP4*. All times are Relative Clock Hour (RCH), showing study day 3 through the end of the 10-day inpatient study.


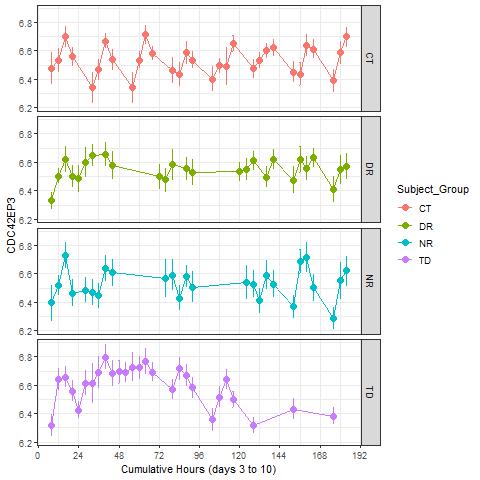


**Figure S9.** Mean (+/- 1 standard error) log2 counts per million gene expression, based on normalized libraries, for the gene *CDC42EP3*. All times are Relative Clock Hour (RCH), showing study day 3 through the end of the 10-day inpatient study.
